# Supplementary material for: Rapid turnover of life-cycle-related genes in the brown algae
Source: Genome Biol. 2019 Feb 14;20:35. doi: 10.1186/s13059-019-1630-6 (PMC6374913; doi:10.1186/s13059-019-1630-6)
Supplement: Supplementary file 3 — Protein alignments, corresponding Gblocks html file and CDS sequences for the lineage conserved gametophyte-biased genes. (ZIP 2109 kb) [file 13059_2019_1630_MOESM3_ESM.zip › Con_GA_alignments/Ec-16_005140.aa_ali.fasta-gb.htm]

Ec-16\_005140.aa.fasta.trans.aa\_ali.fasta


## Gblocks 0.91b Results

Processed file: **Ec-16\_005140.aa.fasta.trans.aa\_ali.fasta**  
Number of sequences: **2**  
Alignment assumed to be: **Protein**  
New number of positions: **623** (selected positions are underlined in blue)

```
                         10        20        30        40        50        60
                 =========+=========+=========+=========+=========+=========+
Ec-16_005140     MKSEVSWSDQVPEREDKPKSNAINDLCFSPDGSKLIVAVESRVLVYDAAEGELVNSLRGH
MSTRG.53519.1    ------------------------------------------------------------
                                                                             


                         70        80        90       100       110       120
                 =========+=========+=========+=========+=========+=========+
Ec-16_005140     KDTVLTVDYAKDGKHFASGGADHTVIIWTSKAEGVLKYTHNESIIRLAYSPGTTKLASCT
MSTRG.53519.1    ------------------------------------------------------------
                                                                             


                        130       140       150       160       170       180
                 =========+=========+=========+=========+=========+=========+
Ec-16_005140     QFDFGLWRQEQKSVVKHKVHAKILSAGWSPDGLHLALGFFDGNVGIRDVNGVEKVTITRK
MSTRG.53519.1    ------------------------------------------------------------
                                                                             


                        190       200       210       220       230       240
                 =========+=========+=========+=========+=========+=========+
Ec-16_005140     TGRCSLWAAGIRRSAFTCSQEGVRLATVCETEAWVWALAGRPGHDELVVGCDGGSIGMHK
MSTRG.53519.1    ------------------------------------------------------------
                                                                             


                        250       260       270       280       290       300
                 =========+=========+=========+=========+=========+=========+
Ec-16_005140     LQFQHVTAIYKDRYAYRENMSDVIVQHLVSEQKVRIKCRDYVEQIAIFRDRLAVRLPDKV
MSTRG.53519.1    ------------------------------------------------------------
                                                                             


                        310       320       330       340       350       360
                 =========+=========+=========+=========+=========+=========+
Ec-16_005140     HVYELTQHDDKYDLRYRLKDKIYLPSAVPSSSSSTLSSPASTISSLAVTSSSRGSESKED
MSTRG.53519.1    ------------------------SSYAKGGSFSSTTS----------------------
                                          #############                      


                        370       380       390       400       410       420
                 =========+=========+=========+=========+=========+=========+
Ec-16_005140     AARHQGSSSLHSQRLSTGSVDGLGGAPRDGDGGGSSFLLVTWNNVVLCEGRVLKLFDFSG
MSTRG.53519.1    ----------------------------------------------------------PX
                                                                             


                        430       440       450       460       470       480
                 =========+=========+=========+=========+=========+=========+
Ec-16_005140     AKVREWVLDAAVRSASVDGGPAGGESLLLGLENGVVLQVFVDNAFPVVLVKASTAAVVGC
MSTRG.53519.1    TKVREWILDAAVRSASVDGGPTGGEALLLGLESGVVLQVFVDNAFPVELVKTGTTAVVCC
                  ###########################################################


                        490       500       510       520       530       540
                 =========+=========+=========+=========+=========+=========+
Ec-16_005140     AMSVHRKKVAVVDAKSRVMVFDVMSKEVEFQETGASSVAFNTSLEDMLCYSGNGKLYVRN
MSTRG.53519.1    AMSVHRKKVAVVDSKRRVIVFDVLSKEVVFQESGARRVAFNSSLEDMLCYSGNGKLYVRA
                 ############################################################


                        550       560       570       580       590       600
                 =========+=========+=========+=========+=========+=========+
Ec-16_005140     GTFPVQEQFIGGEVASYRGARVISLKGVALSTVDVPLTKTVYRHIEARDFQKAHDVACLG
MSTRG.53519.1    GSFPVQEQVIGGEVASYRGARVMSLQGVSLTTIDVPLTKTVYQHIEARDFHKAHAVACLG
                 ############################################################


                        610       620       630       640       650       660
                 =========+=========+=========+=========+=========+=========+
Ec-16_005140     VTQRDWRLLGMSALQAMEFDVSRKAFIRLRDLRFVDILSDIELRTMHEATLLKTNAEARV
MSTRG.53519.1    VTQRDWRILGISAVQTMEFEIGRKAFIRLRDLSFVDVLAEIEIKALQEAALVKSNAETRT
                 ############################################################


                        670       680       690       700       710       720
                 =========+=========+=========+=========+=========+=========+
Ec-16_005140     KMEADSSAELLAHQGNYQGAAKTWARNGMADKAITMFIDLRQWEEAKVFAASSGGSVDAK
MSTRG.53519.1    KIEADASAELLAHQGNYQDAAKTWARNGMSDKAIAMFVDLRQWEEAKVFAASSKGSVDAK
                 ############################################################


                        730       740       750       760       770       780
                 =========+=========+=========+=========+=========+=========+
Ec-16_005140     ELTRRQAEWAEEVGDWSVASELFLKSGEPLRAAEITIKGRGEGWQDALAEIVRSVTKTET
MSTRG.53519.1    ELTRRQAEWAEEVGDWSVASELFLKSGQPLRAAEITIKGKGGGWQDALAEIARSVPKTET
                 ############################################################


                        790       800       810       820       830       840
                 =========+=========+=========+=========+=========+=========+
Ec-16_005140     KILSVCGKELSLAGFDDLAKEAYMKMDDFTNVMALYVKHQNWSEAVKLSEEHGRKYDDAV
MSTRG.53519.1    KILSVCGKELSLAGFDDLAKEAYIKMDDFTNVMALYVKNQNWSEAVKLSEEHDGKFDDCV
                 ############################################################


                        850       860       870       880       890       900
                 =========+=========+=========+=========+=========+=========+
Ec-16_005140     FLPYALWLRDKGRFDEALEAFQRAGRRDLSGAMTEQLILNSVAKARFDDASYYYWRSSTE
MSTRG.53519.1    FLPYALWLRDKGSFDEALEAFRKAGRRDLSGAMTAQLILNAVTKTRFDDASYYYWRSSAE
                 ############################################################


                        910       920       930       940       950       960
                 =========+=========+=========+=========+=========+=========+
Ec-16_005140     AFAAAEKDKTKTSEKDIRKAQRIAEEHSRLADLYHAFHHVHSFTTDPFTDLQPEVLLQVS
MSTRG.53519.1    AYAAA--DDPKNSDKQRREAWRLAAEHSRLADLYHAFHHVHTFTTDPFTDLQPEVLLQVS
                 #####  #####################################################


                        970       980       990      1000      1010      1020
                 =========+=========+=========+=========+=========+=========+
Ec-16_005140     RFLVNSLGEAEPPRGISRAHTLYTLAKQAKVLGAFKLARFAYDRLQRQRVPPLWQDQIDL
MSTRG.53519.1    RFLVNSLGEAEPPRGISRAHTLYTLAKQAKVMGAFKLARFAYDRLQRQRVPPLWQDQIDL
                 ############################################################


                       1030      1040      1050      1060      1070      1080
                 =========+=========+=========+=========+=========+=========+
Ec-16_005140     DMLTVQAKPVRDSPELFAACFRCGASNPLLSPYTGASNSLGSTLSKMPRRTDGGGRGRHD
MSTRG.53519.1    DLLMVQAKPVRDSX----------------------------------------------
                 #############                                               


                       1090      1100      1110      1120      1130      1140
                 =========+=========+=========+=========+=========+=========+
Ec-16_005140     APLPWGDACTTCRHPFVRSFFNFESLPLVEFQPDPGLTDEEALELITASPPQGGRGRGRK
MSTRG.53519.1    ------------------------------------------------------------
                                                                             


                       1150      1160      1170      1180      1190      1200
                 =========+=========+=========+=========+=========+=========+
Ec-16_005140     ARHRGGNQWHEAKEGDADVMALGSDDEGANDDDDLEGNYVPGEELFNRCINRTLEAQEDS
MSTRG.53519.1    ------------------------------------------------------------
                                                                             


                       1210      1220      1230      1240      1250      1260
                 =========+=========+=========+=========+=========+=========+
Ec-16_005140     SEYMVVVASAKCLRALKREDVFRVPPGTRSPRARLYKNMMPDIPLALSQPCGRFFHEEDF
MSTRG.53519.1    ------------------------------------------------------------
                                                                             


                       1270      1280
                 =========+=========+====
Ec-16_005140     EFSYLRESRCPFSRVTDVGDYGSC
MSTRG.53519.1    ------------------------
```

```
Parameters used
Minimum Number Of Sequences For A Conserved Position: 2
Minimum Number Of Sequences For A Flanking Position: 2
Maximum Number Of Contiguous Nonconserved Positions: 8
Minimum Length Of A Block: 10
Allowed Gap Positions: None
Use Similarity Matrices: Yes
```

```
Flank positions of the 3 selected block(s)
Flanks: [326  338]  [422  905]  [908  1033]  

New number of positions in Ec-16_005140.aa.fasta.trans.aa_ali.fasta-gb:  623  (48% of the original 1284 positions)
```
